# Supplementary material for: Dynalign II: common secondary structure prediction for RNA homologs with domain insertions
Source: Nucleic Acids Res. 2014 Nov 21;42(22):13939–48. doi: 10.1093/nar/gku1172 (PMC4267632; doi:10.1093/nar/gku1172)
Supplement: SUPPLEMENTARY DATA [file supp_gku1172_nar-02021-z-2014-File012.zip › manual/javadoc/RNAstructure_java_interface/source/windows/package-frame.html]

RNAstructure\_java\_interface.source.windows


# RNAstructure\_java\_interface.source.windows

## Classes

- DrawingWindow
- DynalignFoldWindow
- DynalignRefoldWindow
- Efn2Window
- FoldDoubleWindow
- FoldSingleWindow
- FoldSuboptimalWindow
- InternalWindow
- MaxExpectWindow
- ModuleWindow
- MultilignWindow
- MultiWindow
- OligoScreenWindow
- OligoWalkWindow
- PartitionDoubleWindow
- PartitionSingleWindow
- ProbKnotWindow
- PseudoknotWindow
- RefoldWindow
- SequenceDisplayWindow
- StochasticWindow
- TurboFoldWindow
